# FGF/FGFR Signaling Coordinates Skull Development by Modulating Magnitude of Morphological Integration: Evidence from Apert Syndrome Mouse Models

**DOI:** 10.1371/journal.pone.0026425

**Published:** 2011-10-28

**Authors:** Neus Martínez-Abadías, Yann Heuzé, Yingli Wang, Ethylin Wang Jabs, Kristina Aldridge, Joan T. Richtsmeier

**Affiliations:** 1 Department of Anthropology, Pennsylvania State University, University Park, Pennsylvania, United States of America; 2 Department of Genetics and Genomic Sciences, Mount Sinai School of Medicine, New York, New York, United States of America; 3 Department of Pathology and Anatomical Sciences, University of Missouri-School of Medicine, Columbia, Missouri, United States of America; University of Massachusetts Medical School, United States of America

## Abstract

The fibroblast growth factor and receptor system (FGF/FGFR) mediates cell communication and pattern formation in many tissue types (e.g., osseous, nervous, vascular). In those craniosynostosis syndromes caused by FGFR1-3 mutations, alteration of signaling in the FGF/FGFR system leads to dysmorphology of the skull, brain and limbs, among other organs. Since this molecular pathway is widely expressed throughout head development, we explore whether and how two specific mutations on *Fgfr2* causing Apert syndrome in humans affect the pattern and level of integration between the facial skeleton and the neurocranium using inbred Apert syndrome mouse models *Fgfr2^+/S252W^* and *Fgfr2^+/P253R^* and their non-mutant littermates at P0. Skull morphological integration (MI), which can reflect developmental interactions among traits by measuring the intensity of statistical associations among them, was assessed using data from microCT images of the skull of Apert syndrome mouse models and 3D geometric morphometric methods. Our results show that mutant Apert syndrome mice share the general pattern of MI with their non-mutant littermates, but the magnitude of integration between and within the facial skeleton and the neurocranium is increased, especially in *Fgfr2^+/S252W^* mice. This indicates that although *Fgfr2* mutations do not disrupt skull MI, FGF/FGFR signaling is a covariance-generating process in skull development that acts as a global factor modulating the intensity of MI. As this pathway evolved early in vertebrate evolution, it may have played a significant role in establishing the patterns of skull MI and coordinating proper skull development.

## Introduction

Fibroblast growth factors (FGFs) and their receptors (FGFRs) play significant roles in vertebrate organogenesis and morphogenesis by controlling levels of cell proliferation, differentiation, migration, adhesion and death [1], [2]. Misregulation can lead to severe dysmorphogenesis and disease, as in the case of the FGFR-related craniosynostosis syndromes (e.g. Apert, Crouzon, Muenke, and Pfeiffer syndromes), which have a prevalence of 6–7 per 100,000 live births and are caused by mutations on FGFR1, 2 and 3. Craniosynostosis syndromes are traditionally characterized by premature fusion of cranial vault sutures, but malformations are widespread, affecting other aspects of the skull, as well as other bodily systems (i.e., brain, limbs, heart, and lung) [3].

More than 98% of cases of Apert syndrome are caused by two dominant mutations involving adjacent amino acids on FGFR2, Ser252Trp (S252W) and Pro253Arg (P253R) [4]. These gain-of-function mutations alter the ligand-binding affinity and specificity of the receptors affecting molecular signaling involved in the development of different tissues including bone [5]. Analysis of the *Fgfr2^+/S252W^* and *Fgfr2^+/P253R^* Apert syndrome mouse models has shown that *Fgfr2* mutations cause primary dysmorphologies of the facial skeleton, the cranial vault, the cranial base, and the brain [6]–[9]. FGF ligands and their receptors are differentially expressed in these cranial regions and tissues mediating cell communication and interaction [10], so it is likely that besides malformations within each structure, altered expression of FGF/FGFR signaling may lead to changes in anatomical relationships among skull regions. Such changes can be evaluated by the comparative analysis of patterns of morphological integration [11].

Morphological integration (MI), assessed by statistical analysis of covariance patterns between phenotypic traits, can reflect genetic, developmental or functional interaction among traits [12]. Biologically, covariation patterns precisely define the interdependence among biological structures. Since MI patterns can facilitate or prevent morphological evolution in certain directions of shape change [12], we propose that conserved MI patterns could constrain clinically relevant shape changes induced by the disease process. The combination of mutation-induced change and conserved MI patterns might provide a mechanism whereby final morphology is altered but maintains a viable and functional shape. Changes in the magnitude of MI alter the degree by which changes in one structure affect other integrated structures [12]. Here we explore the potential role of FGF/FGFR signaling in contributing to patterns and magnitude of skull MI using *Fgfr2^+/S252W^* and *Fgfr2^+/P253R^* Apert syndrome mouse models and their non-mutant littermates.

The critical importance of FGF/FGFR signaling in the appearance of the vertebrate head [13], as well as its role in the development of many cranial tissues prompts us to explore whether the FGF/FGFR pathway is a covariance-generating process at the level of skull phenotypes. The main developmental determinants of the covariation pattern of the mammalian skull are not yet identified and to date few studies have tested whether, how and to what extent the effect of single gene mutations on specific developmental pathways and/or cellular signaling can alter the covariation structure using controlled experimental data [14]–[16]. Our previous analyses showed that despite coronal craniosynostosis in mice carrying *Fgfr2* Apert syndrome mutations, at P0 the facial skeleton is the most affected region of the skull with phenotypic differences between *Fgfr2^+/S252W^* and *Fgfr2^+/P253R^* mutant mice restricted to the posterior aspect of the palate [9]. By exploring how the two *Fgfr2* mutations that cause Apert syndrome in humans affect morphological integration patterns in the mouse skull, we will reveal the relationship between the facial skeleton and the neurocranium in mutant mice relative to their non-mutant littermates and how changes in this interdependence affect skull dysmorphology of Apert syndrome mouse models.

To test whether the two Apert syndrome *Fgfr2* mutations alter the covariation structure of the skulls of mutant mice relative to their non-mutant littermates, and whether the effects of these mutations on MI patterns are similar, we use geometric morphometric analysis of 3D landmark data collected from the newborn (P0) skulls of *Fgfr2^+/S252W^* and *Fgfr2^+/P253R^* Apert syndrome mouse models [6], [7]. Our main goal is not to identify the morphological modules of the mouse skull, but to determine whether the *Fgfr2* Apert syndrome mutations alter a specific pattern of skull MI and thus infer if the FGF/FGFR signaling contributes to patterns of integration of the head. Our null hypothesis (H_o_) is that the skulls of Apert syndrome mouse models (*Fgfr2^+/S252W^* and *Fgfr2^+/P253R^*) and their non-mutant littermates have similar covariance patterns and magnitude of integration. We test this hypothesis through the statistical comparison of covariance patterns and magnitudes within the facial skeleton (face) and neurocranium and between these two anatomical regions. If the null hypothesis cannot be rejected, then FGF/FGFR signaling has no effect on MI patterns. If differences in either the pattern or magnitude of MI are found, then H_o_ is rejected and the alternative hypothesis (H_1_) indicates that the FGF/FGFR signaling affects patterns of MI of the skull. The *Fgfr2* mutations present in these mice could alter the covariation structure in three plausible ways: H_1A_) MI is changed in both pattern and magnitude, resulting in different covariance structures in mutant and non-mutant mouse skulls; H_1B_) MI pattern is maintained but the magnitude is altered resulting in similar patterns but different magnitudes of integration in mutant and non-mutant mice; and H_1C_) MI pattern is altered but magnitude of integration remains unchanged.

## Materials and Methods

### Ethics statement

Mice were generated, euthanized, fixed and imaged in compliance with animal welfare guidelines approved by the Johns Hopkins University, the Mount Sinai School of Medicine, and Pennsylvania State University Animal Care and Use Committees.

### Shape analysis

Our sample consisted of 100 newborn mice bred on C57BL/6J genetic background for 20 generations. Further details on generation of targeting construct have been previously published [6], [7].

**Figure 1:**
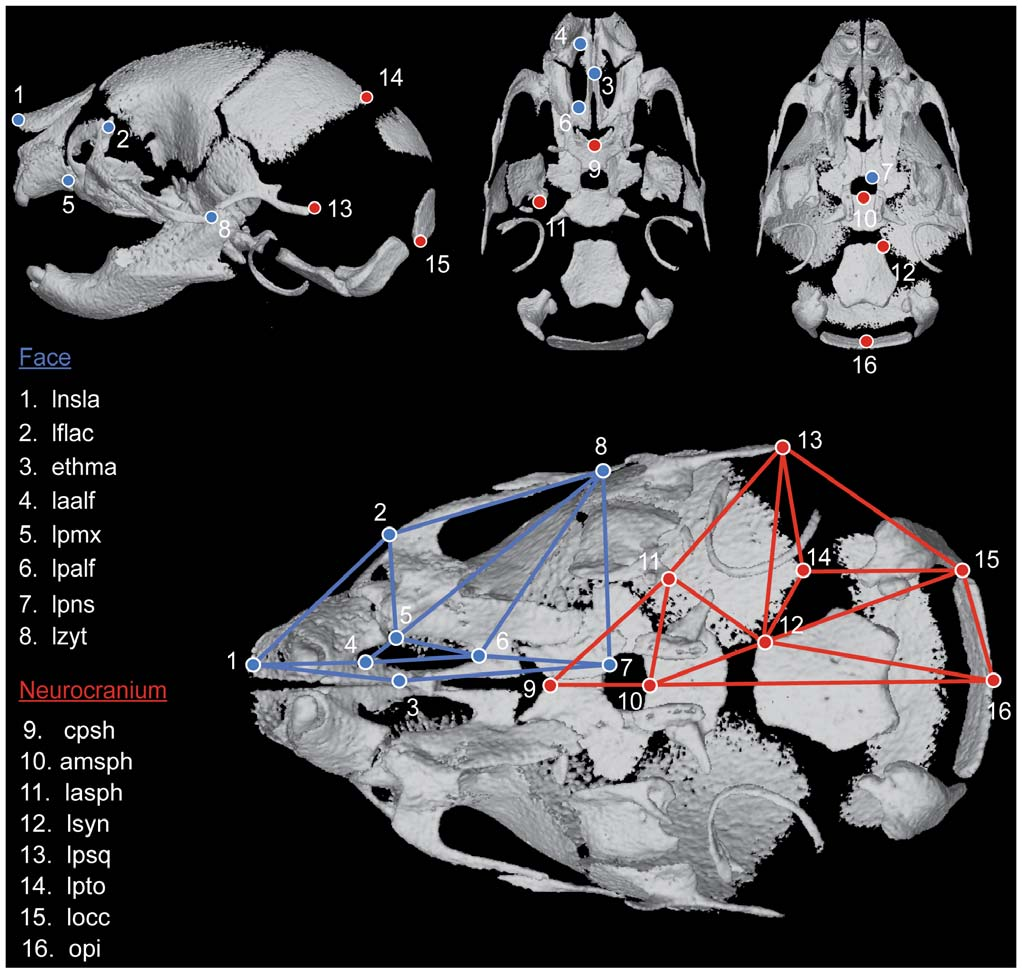
Landmarks collected from μCT reconstructions of P0 mouse skulls. Top: Left lateral view, superior endocranial view with vault removed and inferior view with mandible removed. Bottom: Wireframes used in Fig. 3 to display shape changes of the facial configuration of landmarks (blue) and the neurocranial configuration of landmarks (red). Codes and landmark definitions can be found in Table S1 and at our website http://getahead.psu.edu/LandmarkNewVersion/P0mouseskull_updated_applet.html.

Micro CT (μCT) scans of the heads of *Fgfr2^+/S252W^* and *Fgfr2^+/P253R^* mutant mice and their non-mutant littermates at P0 were acquired by the Center for Quantitative Imaging at the Pennsylvania State University (www.cqi.psu.edu) using the HD-600 OMNI-X high-resolution X-ray computed tomography system (Bio-Imaging Research Inc, Lincolnshire, IL). Pixel sizes range from 0.015 to 0.020 mm, and slice thickness from 0.016 to 0.025 mm. Isosurfaces were reconstructed to represent all cranial bone at P0 using the software package Avizo 6.0 (Visualization Sciences Group, VSG). Based on hydroxyapatite phantoms imaged with the specimens, the minimum thresholds used to create isosurfaces ranged from 70 to 100 mg/cm^3^ partial density of hydroxyapatite. A set of 16 three-dimensional (3D) landmarks from the left side of the skull (Fig. 1) was collected from the isosurfaces. Each specimen was digitized twice by the same observer and measurement error was minimized by averaging the coordinates of the two trials. The maximum accepted measurement error was 0.05 mm. Landmark definitions can be found in Table S1.

To obtain comparable results across groups and across anatomical regions [17], equal sample sizes were used for each group (N = 25) and the shapes of the facial skeleton and the neurocranium were defined using equally sized subsets of mutually exclusive landmarks (K = 8 for each anatomical region) (Fig. 1). Preliminary analyses with a larger number of landmarks, including more landmarks on the rostral aspects of the facial skeleton, the cranial vault and cranial base support the results presented here [18].

### Pattern and magnitude of integration between the facial skeleton and the neurocranium

We quantified the integration between the face and the neurocranium and produced visualizations of the patterns of associated shape changes between these two regions using Two-Block Partial Least Squares analysis (PLS) [19], which performs a singular value decomposition of the covariance matrix. Uncorrelated pairs of new axes are derived as linear combinations of the original variables, with the first pair accounting for the largest amount of inter-block covariation, the second pair for the next largest amount and so on [19]. The amount of covariation is measured by the RV coefficient, which is a multivariate analogue of the squared correlation [20]. Statistical significance was tested using permutation tests under the null hypothesis of complete independence between the two blocks of variables.

Comparison of patterns and magnitude of MI among *Fgfr2^+/S252W^* and *Fgfr2^+/P253R^* mutant mice and their non-mutant littermates required PLS analyses of varying subsets of individuals including: 1) all mutant and non-mutant mice, 2) only non-mutant mice, 3) only mutant mice, 4) *Fgfr2^+/S252W^* mutant mice and their non-mutant littermates, 5) *Fgfr2^+/P253R^* mutant mice and their non-mutant littermates. Each PLS analysis was applied to the adjusted coordinate data obtained from two separate Procrustes fits (one for the facial landmarks and another for the neurocranial landmarks). Procrustes fits were performed separately for each PLS analysis. General Procrustes Analysis (GPA) is a procedure that superimposes configurations of landmarks by shifting them to a common position, rotating and scaling them to a standard size until a best fit of corresponding landmarks is achieved [21]. Because the covariance matrix as estimated by GPA is neither invariant nor identifiable [22] we have estimated the covariance of the face and the neurocranium separately, and together as a composite structure, enabling an internal check of the general results of our analyses using varying sets of landmarks in additional analyses shown in Supporting Information S1.

As size affects the whole skull and can inflate measures of integration [20], we explored the effect of allometry by computing a multivariate regression of shape [23] on centroid size, measured as the square root of the summed distances between each landmark coordinate and the centroid of the landmark configuration [21]. To adjust for size-shape differences we repeated all the PLS analyses using the residuals of the multivariate regression.

To statistically compare the covariance matrices across *Fgfr2^+/S252W^* and *Fgfr2^+/P253R^* Apert syndrome mouse models and their non-mutant littermates, we computed two-by-two matrix correlation tests between the covariance matrices of: 1) *Fgfr2^+/+^* non-mutant mice of both models; 2) *Fgfr2^+/S252W^* and *Fgfr2^+/P253R^* mutant mice; 3) *Fgfr2^+/S252W^* and their non-mutant littermates, 4) *Fgfr2^+/P253R^* mutant mice and their non-mutant littermates. For each analysis, a matrix permutation test against the null hypothesis of complete dissimilarity of the covariance matrices was performed by permuting landmarks and including the diagonals of the covariance matrices after 10,000 randomization rounds. All analyses were performed using MorphoJ [24].

Finally, we computed the variance of the eigenvalues (EV) [25] as an alternative metric of overall integration between the face and neurocranium, as well as to quantify and compare the magnitude of integration within the face and the neurocranium across mouse groups. In poorly integrated structures where correlations between variables are weak, variance will be distributed across many eigenvectors, resulting in low EV. Conversely, in highly integrated structures where correlations are high, variance will be concentrated in few eigenvectors, resulting in high EV scores [25]. We computed the variance-covariance matrices of the Procrustes coordinates of the face (K = 8), the neurocranium (K = 8) and the whole skull (face+neurocranium, K = 16) for each group separately. From each variance-covariance matrix we computed the eigenvalues and obtained ranges of EV integration values by resampling each dataset with replacement for 1,000 iterations. To compare the integration measures across groups and remove variation in the index caused by the magnitude of the overall variance we standardized the EV scores by the total shape variance within the entire sample group following Young [26]. These procedures were calculated using the PopTools plug-in for Excel version 3.2 (http://www.poptools.org).

## Results

### Patterns of skull MI are not disrupted by Apert syndrome *Fgfr2* mutations

**Figure 2:**
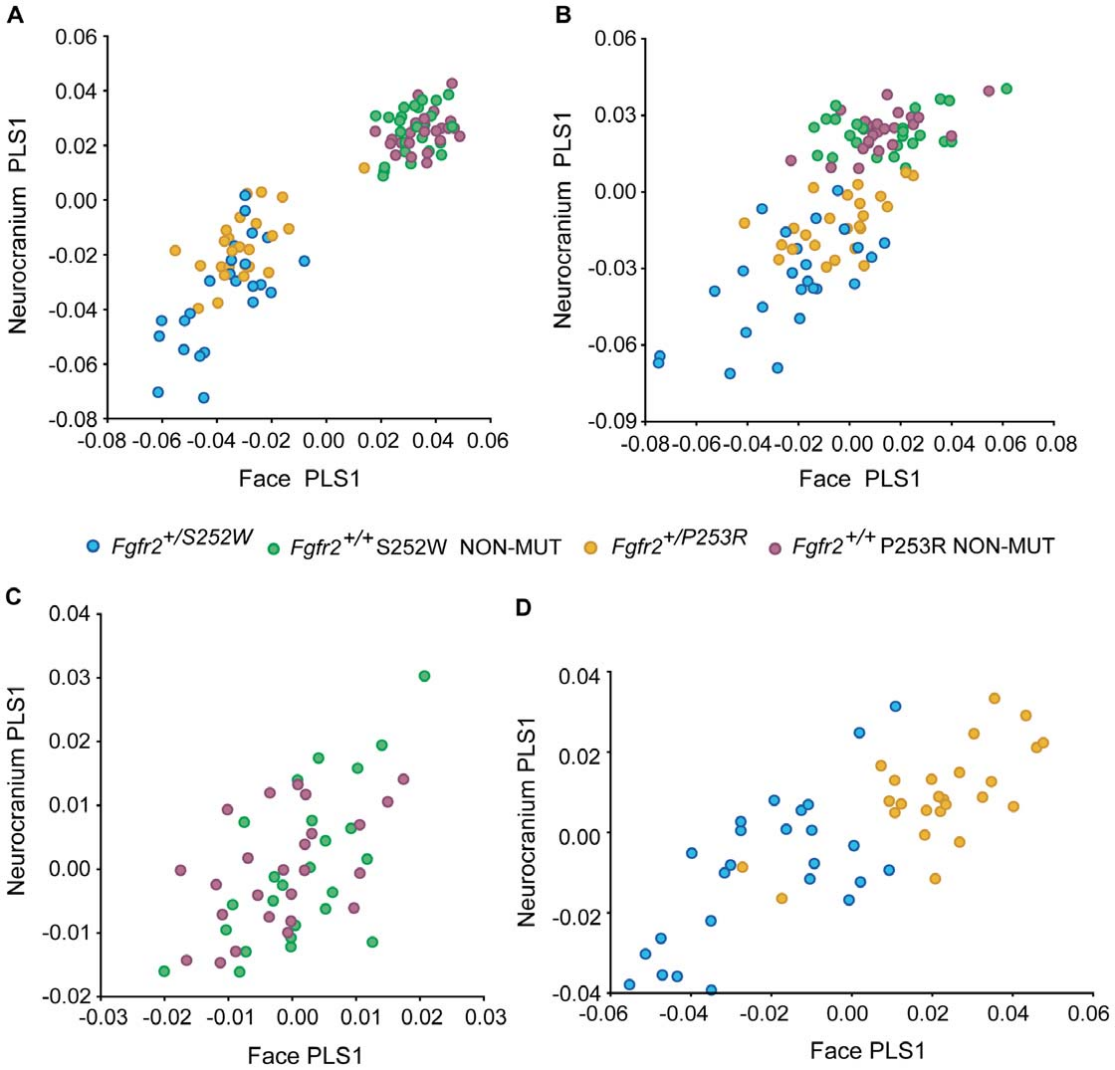
Scatterplots of PLS1 scores of the facial and the neurocranial skeleton using varying subsets of individuals. A) PLS analysis including Apert syndrome mouse models and non-mutant littermates before adjusting for allometry; B) PLS analysis including Apert syndrome mouse models and non-mutant littermates after adjusting for allometry; C) PLS analysis of non-mutant littermates after adjusting for allometry; D) PLS analysis of mutant Apert syndrome mouse models after adjusting for allometry.

The pooled PLS analysis including all mice was used to test whether Apert syndrome *Fgfr2* mutations alter the normal pattern of MI within the skull. Results showed that the first pair of PLS axes (PLS1) explains 96.2% of the total covariance (Fig. 2A) and that the overall strength of association between the facial skeleton and the neurocranium is high (RV = 0.72) and statistically significant (Table 1). Distribution of specimens along the first pair of PLS axes displays a separation into two groups, reflecting the morphological differences between mice carrying the *Fgfr2* mutations (negative PLS1 values) and their non-mutant littermates (positive PLS1 values) (Fig. 2A). Non-mutant littermates from both models completely overlap, whereas *Fgfr2^+/S252W^* and *Fgfr2^+/P253R^* mutant mice only partially overlap, with *Fgfr2^+/S252W^* mutant mice showing an extended range of covariation (Fig. 2A).

**Table 1:** Results of PLS analyses.

Samples used	RV coefficient	P-value	% Total Cov PLS1	Corr PLS1	P-value
All groups	0.72 (0.39)	0.0001 (0.0001)	96.2% (90.3%)	0.92 (0.75)	0.0001 (0.0001)
Both NON-MUT	0.19 (0.16)	0.0364 (0.3312)	56.9% (34.7%)	0.60 (0.57)	0.0314 (0.5745)
Both MUT	0.40 (0.36)	0.0001 (0.0001)	70.0% (69.4%)	0.82 (0.77)	0.0001 (0.0001)
252 NON-MUT/MUT	0.83 (0.51)	0.0001 (0.0001)	96.2% (91.4%)	0.94 (0.74)	0.0001 (0.0001)
253 NON-MUT/MUT	0.71 (0.40)	0.0001 (0.0001)	97.1% (84.8%)	0.93 (0.77)	0.0001 (0.0001)

For each grouping we provide the RV coefficient of overall integration between the face and the neurocranium and associated P-value; the percentage of total covariation explained by PLS1 axes; the correlation score between facial and neurocranial PLS1 scores and associated P-value. Values in parentheses correspond to results obtained after adjusting for allometric effects.

The multivariate regression of skull shape on centroid size showed that size significantly predicts 29.33% of total shape variation (P-value<0.0001). The separation between mutant and non-mutant mice disappears (Fig. 2B) and the RV coefficient is reduced (RV = 0.39; Table 1) when allometry is adjusted for, indicating that size is a common factor affecting the facial skeleton and the neurocranium thereby inflating integration measures. Nevertheless, PLS1 still explains a very high percentage of the total covariance (90.3%; Table 1), showing that a single pair of PLS axes, shared by mice carrying these *Fgfr2* Apert syndrome mutations and their non-mutant littermates, explains nearly all covariation between the facial skeleton and the neurocranium. This indicates that Apert syndrome mouse models and their non-mutant littermates have similar covariance patterns.

The matrix correlation analyses confirmed that *Fgfr2^+/S252W^* and *Fgfr2^+/P253R^* mutant mice and their non-mutant littermates have similar covariation structures. The matrix correlation values obtained between groups were: 1) *Fgfr2^+/+^* non-mutant mice of both models: r = 0.69, p<0.0001; 2) *Fgfr2^+/S252W^* and *Fgfr2^+/P253R^* mutant mice: r = 0.49, p<0.0001; 3) *Fgfr2^+/S252W^* and their non-mutant littermates: r = 0.60, p<0.0001; 4) *Fgfr2^+/P253R^* mutant mice and their non-mutant littermates: r = 0.47, p<0.0001. The two-by-two comparisons of the covariance matrices always provided high and significant matrix correlation values, and therefore the statistical null hypothesis of complete dissimilarity between covariance matrices was rejected, providing further support for similarity of covariance matrices between Apert syndrome mouse models and their non-mutant littermates.

### Similar pattern but increased magnitude of skull MI in Apert syndrome mouse models

To determine whether the FGF/FGFR signalling pathway modulates the intensity of established patterns of skull integration, we compared the magnitude of MI between face and neurocranium by comparing the RV coefficient of the PLS analysis of non-mutant mice with the RV coefficient of the PLS estimated for mutant mice.

Scatterplots of the PLS analysis of non-mutant littermates from both models show complete overlap before (data not shown) and after removing allometry (Fig. 2C). Size significantly predicted 10.79% of shape variation (P-value<0.0001) and the PLS results showed that the total percentage of covariation between the face and the neurocranium is moderate in non-mutant mice (34.7%; Table 1). The PLS analysis of non-mutant mice provided a low but significant measure of overall integration between the face and the neurocranium (RV = 0.19). When allometry was statistically removed, the RV was reduced and no longer significant, indicating some degree of independence between the face and the neurocranium in non-mutant mice (Table 1). However, modularity testing of the face and the neurocranium in non-mutant mice did not confirm this result (Supporting Information S1 and Fig. S1).

The PLS of *Fgfr2^+/S252W^* and *Fgfr2^+/P253R^* mice reveal two separate clusters that are distributed along the same PLS1 axes (Fig. 2D). Size only predicted 3.41% of total shape variation (P-value = 0.12) in mutant Apert syndrome mice and the PLS results were very similar before (data not shown) and after removing allometry (Fig. 2D), showing a high total percentage of covariation between the facial skeleton and the neurocranium (70%; Table 1). The RV coefficient estimated for the mutant mice was twice as large (RV = 0.40) as that estimated for the non-mutant littermates (Table 1). Though the covariance patterns of Apert syndrome mouse models and their non-mutant littermates are similar, our results show that Apert syndrome mouse models differ from their non-mutant littermates in magnitude of MI, which leads to rejection of H_o_ and provides support for H_1B_.

### Apert syndrome *Fgfr2* mutations alter skull MI with differing magnitude

**Figure 3:**
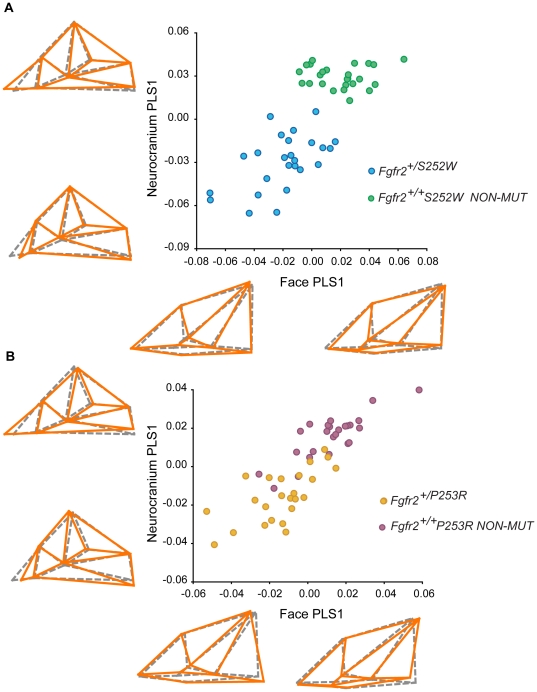
PLS analyses among each Apert syndrome mouse model and their non-mutant littermates after removing allometry. A) *Fgfr2^+/S252W^* and non-mutant littermates; B) *Fgfr2^+/P253R^* and non-mutant littermates. Associated facial and neurocranial shape changes corresponding to the first pair of PLS1 axes show similar skull MI patterns between the two models. Orange wireframes display face and neurocranium shape changes associated with positive and negative values of PLS1 in comparison to mean shape PLS1 values (grey dashed wireframe). For anatomical correspondence see Fig. 1. Note that all landmarks cannot be seen from a single skull view and we chose to display the inferior view of the skull because main shape changes occur in the palate and the anterior aspect of the neurocranium.

Two separate PLS analyses, each comparing one of the mutant groups (*Fgfr2^+/S252W^* and *Fgfr2^+/P253R^*) with their respective non-mutant littermates, were used to determine the effect of each mutation on normal patterns of morphological integration (Fig. 3A, B). Shape patterns of MI between the facial skeleton and the neurocranium are very similar in *Fgfr2^+/S252W^* and *Fgfr2^+/P253R^* mutant mice relative to their respective non-mutant littermates (Fig. 3A, B). In both Apert syndrome mouse models (negative PLS1 values) and their non-mutant littermates (positive PLS1 values), covariation between the face and the neurocranium is mainly driven by associated changes in the posterior aspects of the facial skeleton and the palate and the anterior cranial base (Fig. 3A, B). In *Fgfr2^+/S252W^* and *Fgfr2^+/P253R^* mutant mice, the zygomatic bone is located more anteriorly and the posterior edge of the horizontal plate of the palatine bone is shifted postero-medially. The associated neurocranial change is a posterior shift in the position of the presphenoid that affects cranial base flexion and the relative positioning of the facial skeleton. Moreover, in mutant Apert syndrome mouse models the neurocranium is shortened and widened, resulting in the typical brachycephalic shape traditionally associated with premature closure of the coronal suture.

Both PLS analyses showed that the first pair of PLS axes (PLS1) explained a high percentage of total covariation and that RV coefficients were high and significant (Table 1, Fig. 3A, B). However, the magnitude of association between the face and neurocranium in *Fgfr2^+/S252W^* mutant mice and their non-mutant littermates is higher (RV = 0.51) relative to that of the *Fgfr2^+/P253R^* mutant mice and their non-mutant littermates (RV = 0.40). Also, *Fgfr2^+/S252W^* mutant mice and their non-mutant littermates are clearly differentiated along the first pair of PLS1 axes showing no overlap (Fig. 3A), whereas *Fgfr2^+/P253R^* mutant mice and their non-mutant littermates overlap with individuals continuously distributed along the first pair of PLS1 axes (Fig. 3B). Analyses including the allometric components of shape provided very similar results (data not shown).

### Increased integration within and between the facial skeleton and the neurocranium in Apert syndrome mouse models, especially in *Fgfr2^+/S252W^*

**Figure 4:**
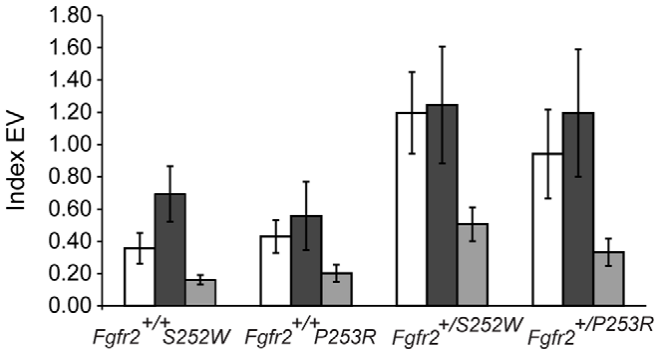
Comparison of MI within and between facial skeleton and neurocranium across Apert syndrome mouse models and non-mutant littermates. Bar graphs with standard deviation error bars comparing the distribution of the integration index (EV, Eigenvalue variance standardized by group variance*10^5^) within the face (white bars), within the neurocranium (dark grey bars) and between the face and the neurocranium (light grey bars).

The variance of the eigenvalues confirmed that the pattern of MI is not disrupted in mutant Apert syndrome mouse models in comparison with their non-mutant littermates. Analyses of all four groups show that of the two regions the neurocranium is the most integrated; and that the integration within regions is higher than integration between regions. This analysis also indicates that the magnitude of MI within and between regions is increased in mice carrying the *Fgfr2* mutations, especially the S252W mutation (Fig. 4), confirming all previous results and supporting H_1B_.

## Discussion

Extensive research has shown that cranial integration is highly conserved across placental mammals [27], [28] and that this shared pattern of skull covariation can be preserved even under genetic and developmental alterations [29], [30], disease [31], and cultural deformation practices [32]. That MI of the vertebrate skull is strongly conserved is emphasized by the finding that differences in cranial shape across species and populations are more commonly associated with changes in magnitude rather than in pattern of MI [28]. Our results confirm that despite severe craniofacial dysmorphologies, mice carrying Apert syndrome *Fgfr2* mutations share MI patterns with their non-mutant littermates, but show increased integration (Table 1 and Figs. 2, 3, 4), providing evidence to reject Ho and support H_1B_. Thus, our results indicate that the FGF/FGFR signaling system may contribute to the covariance-generating processes operating within skull development by adjusting the magnitude of MI patterns.

### FGF/FGFR signaling: a global factor modulating skull morphological integration

Our analysis provides another type of evidence for the important role of FGF/FGFR signaling pathways as a global factor in coordinating skull growth. FGF/FGFR signaling interacts with other major signaling pathways that regulate chondrogenesis and osteogenesis [1] and directly affects osteoprogenitor, osteogenic, as well as other cell types. The changes induced by FGF/FGFR signaling (e.g., differentiation, proliferation, adhesion, apoptosis) have significant effects on the morphology of developing bone, but also have a significant global influence on proper coordination among different regions of the growing skull. Recently it has been shown that alteration of FGF signaling for somitogenesis during gastrulation in early vertebrates may have led to the creation of an anterior region with unsegmented paraxial mesoderm and the appearance of the “new head” [13]. Our results add additional evidence to the key role of FGF/FGFR signaling in the development of the skull and suggest a potential role in the maintenance of established MI patterns in the evolution of the vertebrate head.

Altered balance of FGF/FGFR signaling in Apert syndrome mouse models not only produces widespread primary dysmorphologies [6]–[9], but also modification of the magnitude of integration among cranial structures (Table 1, Fig. 4), which may explain further secondary dysmorphogenesis in craniosynostosis syndromes. In our Apert syndrome mouse models we have found that both the facial skeleton and the neurocranium are primarily affected by the *Fgfr2* mutations [9], and that the MI patterns are conserved, but the magnitude of integration between the face and the neurocranium is increased. Preliminary analyses based on reduced samples of Apert syndrome mouse models at postnatal day 2 indicate that this tendency is maintained and the magnitude of integration between the facial skeleton and neurocranium is even greater at later stages of development (data not shown). This indicates that alteration of FGF/FGFR signaling leads to skull dysmorphologies that could represent the combined result of primary shape changes caused by the direct effects of the Apert syndrome mutations and secondary shape changes triggered by the indirect effect of increased covariation between the face and the neurocranium.

The anatomical separation of the skull into facial skeleton and neurocranium mainly reveals functional interactions within regions (i.e. the facial skeletal morphology develops and responds to visual, olfactory and masticatory soft tissues and functions, whereas the neurocranium mainly responds to CNS growth and vascularization while functioning to protect the brain). Our results indicate that the face and the neurocranium have a simply structured covariation pattern that can be summarized into a single pair of PLS axes that account for more than 90% of the total covariation (Table 1) that is shared across *Fgfr2^+/S252W^* and *Fgfr2^+/P253R^* mutant mice and their non-mutant littermates (Fig. 2). As expected for modular structures [12], integration within the facial skeleton and within the neurocranium is higher than integration between regions (Fig. 4). However, a priori hypothesis testing that face and neurocranium represent two different modules was not supported by our analysis (Fig. S1 and Supporting Information S1).

Covariation analyses of skull regions defined by alternative developmental criteria to partition the skull, such as the embryological origin (neural crest/mesoderm derived bones) or the mode of ossification (endochondral/intramembranous ossification) did not reveal any significant effects of FGF/FGFR alteration on patterns of skull morphological integration in *Fgfr2^+/S252W^* and *Fgfr2^+/P253R^* Apert syndrome mouse models (Supporting Information S2).

### Molecular underpinnings of increased skull MI in Apert syndrome mouse models

The specific molecular interactions that lead to increased skull MI within and between the facial skeleton and the neurocranium of *Fgfr2^+/S252W^* and *Fgfr2^+/P253R^* mice (Fig. 4) are difficult to predict because FGF/FGFR signaling can be directly and indirectly up- and down regulated by other interacting signaling pathways (i.e., BMP, MAPK, Wnt, Ihh, Shh) [1]. However, knowledge of the overall effect of *Fgfr2* mutations on FGF/FGFR signaling can provide some clues and help to formulate hypotheses. Our Apert syndrome mouse models are heterozygotes, so cells expressing Fgfr2 receptors have both normal and mutant receptors, the latter of which lead to aberrant Fgfr2 activation by modifying ligand affinity and specificity [5]. If mutant receptors are homogeneously distributed throughout the facial and neurocranial regions of the skulls of Apert syndrome mouse models, a net increase in the length and strength of Fgfr2 signaling may be responsible for the increased level of integration within the face, within the neurocranium, as well as between the face and the neurocranium.

Overall, the two *Fgfr2* Apert syndrome mutations produce similar morphological effects [6]–[9]. However, significant localized differences have been reported between the two models and the S252W mutation has been associated with more severe skull dysmorphologies [9]. Here we provide an additional piece of evidence of the difference in the effects of the two mutations on skull development, showing that the S252W mutation increases the range of covariation (Fig. 2A, B) and the magnitude of integration within and between the facial skeleton and the neurocranium (Fig. 4). Since each mutation alters the Fgfr2 receptor differently [33]–[36], morphological differences between *Fgfr2^+/S252W^* and *Fgfr2^+/P253R^* Apert syndrome mouse models may stem from differential affinity of the mutated receptors for specific ligands [35]. In fact, the distinct nature of the gain of function contacts mediated by the S252W and P253R mutations has been proposed to reflect the phenotypic variability between the two mutation subsets of Apert syndrome patients and mice [34]–[36]. Differences in the ligand binding between the two *Fgfr2* mutations and specificity of their gain of function in particular tissues might also underlie the different effects on range of covariation and the magnitude of integration between and within the face and the neurocranium demonstrated here. For instance, the increased affinity of the S252W mutation for Fgf2, which at E14.5 is widely expressed in the cranial vault and cranial base but especially in facial bone and limbs (www.genepaint.org), may explain why the most intense dysmorphic effects are concentrated on the face and why overall integration between the face and the neurocranium is more increased in *Fgfr2^+/S252W^* mutant mice (Fig. 4). In fact, *Fgfr2^+/S252W^* mutant mice show the most substantial increase of integration within the face and display the palatal dysmorphologies that are more frequently associated with Apert syndrome patients carrying the S252W mutation [9]. In comparison to the S252W mutation that enhances signaling with a limited subset of Fgfs, the P253R mutation enhances signaling with many Fgfs [35] and one hypothesis is that the P253R mutation may respond to many more signals that can have overlapping, redundant, and/or counteracting effects, resulting in a more moderate increase of MI.

### Conclusions

In FGFR1-3 related craniosynostosis syndromes, balance alteration of cell biological activities regulated by FGF/FGFR signaling causes changes in molecular spatio-temporal dynamics leading to anomalies in cellular and developmental processes that change the shape of skull bones, but the structure of the osseous tissue remains within normal limits. Here we show that besides morphological dysmorphologies (premature suture fusion, midfacial hypoplasia, and cleft palate), Apert syndrome *Fgfr2* mutations affect morphological integration patterns within the skull by increasing its magnitude. As FGFs and their receptors are expressed in other developing tissues such as peripheral nerves, the CNS and vasculature, it is likely that FGF/FGFR signaling also contributes to the integration within each of these structures, as well as between them during development of the head. We propose that cell communication and cell interactions that are influenced by FGF/FGFR signaling underlie basic developmental processes coordinating head morphogenesis and contribute to the coordinated growth and development of a functional and operational head.

## Supporting Information

Figure S1 Histograms of the distribution of the RV coefficients computed after all possible random partitions of equally sized subsets of landmarks (K = 8). Arrows indicate the RV coefficient for the actual hypothesis tested (modularity of face and neurocranium) in each grouping of samples: *Fgfr2^+/+^* non-mutant mice of both models (white); *Fgfr2^+/S252W^* and *Fgfr2^+/P253R^* mutant mice (black); *Fgfr2^+/S252W^* (purple) and *Fgfr2^+/P253R^* (yellow) Apert mice.
(TIF)

Table S1 Anatomical definitions of 16 three-dimensional skull landmarks collected from μCT images of mice at P0. Landmarks are illustrated in Fig. 1.
(DOC)

Supporting Information S1 Modularity test: facial and neurocranial skeleton within the skull of *Fgfr2^+/S252W^* and *Fgfr2^+/P253R^* Apert syndrome mouse models and their non-mutant littermates.
(DOC)

Supporting Information S2 Morphological integration patterns between 1) neural crest/mesoderm derived bones and 2) endochondral/intramembranous bones.
(DOC)
