# Supplementary figures and images for: FGF/FGFR Signaling Coordinates Skull Development by Modulating Magnitude of Morphological Integration: Evidence from Apert Syndrome Mouse Models
Source: PLoS One. 2011 Oct 28;6(10):e26425. doi: 10.1371/journal.pone.0026425 (PMC3203899; doi:10.1371/journal.pone.0026425)

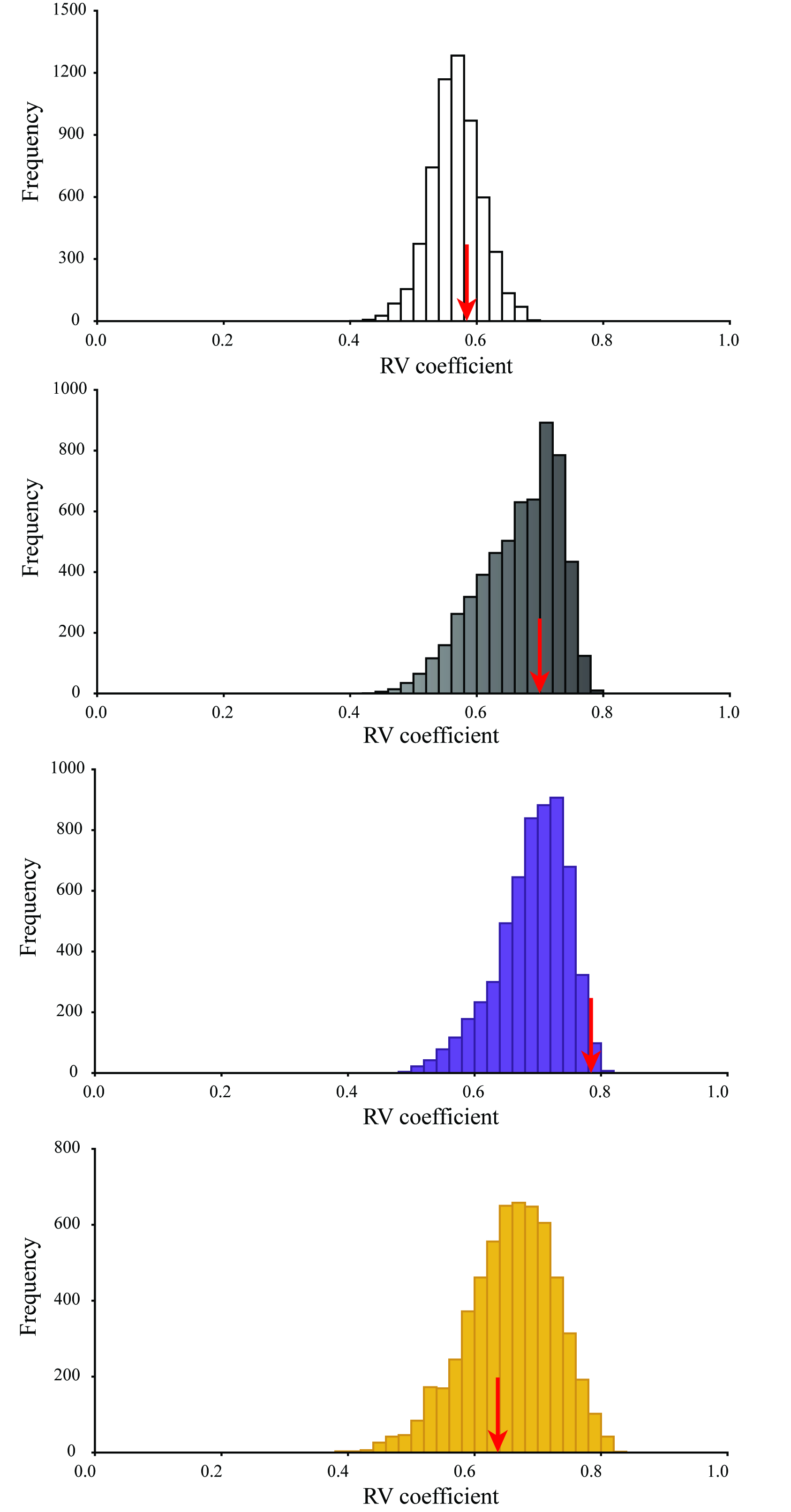

Supplement: Figure S1 — Histograms of the distribution of the RV coefficients computed after all possible random partitions of equally sized subsets of landmarks (K = 8). Arrows indicate the RV coefficient for the actual hypothesis tested (modularity of face and neurocranium) in each grouping of samples: Fgfr2+/+ non-mutant mice of both models (white); Fgfr2+/S252W and Fgfr2+/P253R mutant mice (black); Fgfr2+/S252W (purple) and Fgfr2+/P253R (yellow) Apert mice. (TIF) [file pone.0026425.s001.tif]
