# Supplementary material for: FGF/FGFR Signaling Coordinates Skull Development by Modulating Magnitude of Morphological Integration: Evidence from Apert Syndrome Mouse Models
Source: PLoS One. 2011 Oct 28;6(10):e26425. doi: 10.1371/journal.pone.0026425 (PMC3203899; doi:10.1371/journal.pone.0026425)
Supplement: Table S1 — Anatomical definitions of 16 three-dimensional skull landmarks collected from μCT images of mice at P0. Landmarks are illustrated in Fig. 1. (DOC) [file pone.0026425.s002.doc]

| **Landmark** | **Code** | **Anatomical definition** |
| --- | --- | --- |
| 1 | lnsla | Most antero-medial point of the nasal bone, left |
| 2 | lflac | Intersection of frontal process of maxilla with frontal and lacrimal bones, left |
| 3 | ethma | Most antero-superior point of the intersection of the left and right anterior turbinates |
| 4 | laalf | Most anterior point of the anterior palatine foramen, left |
| 5 | lpmx | Most infero-lateral point of the premaxillary-maxillary suture, taken on premaxilla, left |
| 6 | lpalf | Most posterior point of the anterior palatine foramen, left |
| 7 | lpns | Most antero-lateral indentation at the posterior edge of the horizontal plate of the palatine bone , left |
| 8 | lzyt | Intersection of zygoma with zygomatic process of temporal, taken on zygoma, left |
| 9 | cpsh | Most anterior point of the indentation in the center of the presphenoid |
| 10 | amsph | Most antero-medial point on the body of the sphenoid |
| 11 | lasph | Postero-medial point of the inferior portion of the alisphenoid, left |
| 12 | lsyn | Most antero-lateral point on corner of the basioccipital at the basi occipital synchondrosis, left |
| 13 | lpsq | Most posterior point on the posterior extension of the forming squamosal, left |
| 14 | lpto | Most postero-medial point on the parietal, left |
| 15 | locc | Most infero-lateral point on the squamous occipital, left |
| 16 | opi | Mid-point on the posterior margin of the foramen magnum, taken on basioccipital |
