# Supplementary material for: FGF/FGFR Signaling Coordinates Skull Development by Modulating Magnitude of Morphological Integration: Evidence from Apert Syndrome Mouse Models
Source: PLoS One. 2011 Oct 28;6(10):e26425. doi: 10.1371/journal.pone.0026425 (PMC3203899; doi:10.1371/journal.pone.0026425)
Supplement: Supporting Information S1 — Modularity test: facial and neurocranial skeleton within the skull of Fgfr2+/S252W and Fgfr2+/P253R Apert syndrome mouse models and their non-mutant littermates. (DOC) [file pone.0026425.s003.doc]

**SUPPORTING INFORMATION S1**

We tested the *a priori* hypothesis that the facial skeleton and the neurocranium are modules of the skull of *Fgfr2^+/S252W^* and *Fgfr2^+/P253R^* mutant mice and their non-mutant littermates using the method described by Klingenberg (2009). This method is based on a simultaneous-fit approach, in which a single Procrustes superimposition is performed to fit simultaneously the landmarks from the facial and neurocranial regions, and thus includes in the analysis information about the connection between the two modules. If the facial skeleton (face) and the neurocranium represent true modules, these two cranial regions should be statistically highly integrated within themselves and relatively independent from each other. To estimate the strength of the association between the face and the neurocranium in mutant and non-mutant mice from each group, we measured the covariation between the two sets of Procrustes fitted landmarks using the RV coefficient (Klingenberg, 2009), which is a multivariate analogue of the squared correlation. If our partition of the skull into facial and neurocranial anatomical regions reflects the boundaries of modules within the mouse skull, we expect a lower association between the facial and neurocranial subsets than any other random partitions of the landmarks. A higher RV coefficient indicates that the face and the neurocranium are not independent but integrated.

The null hypothesis of complete independence between modules is statistically evaluated through the use of a permutation test that computes the pairwise RV coefficients for all possible alternative partitions between equally sized subsets of landmarks (Klingenberg, 2009). The significance level is estimated as the proportion of times in which an alternative partition provided an equal or higher RV coefficient than the RV value obtained using the original partition.

Facial skeleton and neurocranium are not modules

None of the modularity tests provided significant RV coefficients (*Fgfr2^+/+^* non-mutant mice of both models: RV=0.58, p-value=0.68; both *Fgfr2^+/S252W^* and *Fgfr2^+/P253R^* mutant mice: RV=0.69, p-value=0.61; *Fgfr2^+/S252W^* mutant mice; RV=0.78, p-value=0.98; *Fgfr2^+/S252W^* mutant mice: RV=0.63, p-value=0.35). Therefore, in Apert syndrome mouse models and non-mutant littermates the face and the neurocranium do not show a level of independence expected for modules. Also, our results showed that the integration within the face and the neurocranium measured by the RV coefficient was higher within Apert syndrome mouse models than within their non-mutant littermates (Fig. S1). Comparing the two Apert syndrome mouse models, the *Fgfr2^+/S252W^* mutant mice showed a higher integration than *Fgfr2^+/P253R^* mutant mice (Fig. S1), confirming all the results previously reported in the main manuscript (Table 1, Figs 2-4).
