# Supplementary material for: FGF/FGFR Signaling Coordinates Skull Development by Modulating Magnitude of Morphological Integration: Evidence from Apert Syndrome Mouse Models
Source: PLoS One. 2011 Oct 28;6(10):e26425. doi: 10.1371/journal.pone.0026425 (PMC3203899; doi:10.1371/journal.pone.0026425)
Supplement: Supporting Information S2 — Morphological integration patterns between 1) neural crest/mesoderm derived bones and 2) endochondral/intramembranous bones. (DOC) [file pone.0026425.s004.doc]

**SUPPORTING INFORMATION S2**

To explore whether other developmental factors contributed similarly to skull integration patterns in *Fgfr2^+/S252W^* and *Fgfr2^+/P253R^* Apert syndrome mutant and their non-mutant littermates, we analyzed patterns of MI using cranial regions defined on the basis of alternative criteria: 1) the embryological origin of osteogenic cells (neural crest/mesoderm), and 2) the mode of ossification of specific bones (endochondral/intramembranous).

**1) Neural-crest/mesoderm**

We defined two subsets of mutually exclusive landmarks based on whether the landmarks were located on bones derived from neural crest cells or mesoderm cells [1,2], as shown in Table S2.

Table S2. Landmark distribution into neural crest and mesoderm derived regions. More detailed information at <http://getahead.psu.edu/LandmarkNewVersion/P0mouseskull_updated_applet.html>

| **Code** | **Neural crest** | **Mesoderm** | **Definition** |
| --- | --- | --- | --- |
| amsph | 1 |  | Most antero-medial point on the body of the sphenoid |
| ethma | 2 |  | Most antero-superior point of the intersection of the left and right anterior turbinates |
| lasph | 3 |  | Postero-medial point of the inferior portion of the alisphenoid, left |
| lflac | 4 |  | Intersection of frontal process of maxilla with frontal and lacrimal bones, left |
| lnsla | 5 |  | Most antero-medial point of the nasal bone, left |
| lpns | 6 |  | Most antero-lateral indentation at the posterior edge of the horizontal plate of the palatine bone, left |
| lpsq | 7 |  | Most posterior point on the posterior extension of the forming squamosal, left |
| lzyt | 8 |  | Intersection of zygoma with zygomatic process of temporal, taken on zygoma, left |
| bas |  | 1 | Mid-point on the anterior margin of the foramen magnum, taken on basioccipital |
| locc |  | 2 | Most infero-lateral point on the squamous occipital, left |
| loci |  | 3 | Superior posterior point on the ectocranial surface of occipital lateralis on the foramen magnum, left |
| lpto |  | 4 | Most postero-medial point on the parietal, left |
| lsyn |  | 5 | Most antero-lateral point on corner of the basioccipital at the basi occipital synchondrosis, left |
| opi |  | 6 | Mid-point on the posterior margin of the foramen magnum |

Using these subsets of neural crest and mesoderm landmarks, we performed PLS analyses pooling all mice (*Fgfr2^+/S252W^* and *Fgfr2^+/P253R^* Apert syndrome mutant mice and their non-mutant littermates) before and after adjusting for allometry. The scatterplots of the PLS analyses (Fig. S2) showed that *Fgfr2^+/S252W^* and *Fgfr2^+/P253R^* mutant mice share the first PLS axes of covariation between the neural crest and mesoderm derived regions with their non-mutant littermates, but specimens showed a dispersed distribution along PLS1 axes (Fig. S2).

Figure S2. Scatterplot of the PLS1 scores of the neural crest and mesoderm derived regions of the skull before (A) and after (B) removing allometry and including all *Fgfr2^+/S252W^* and *Fgfr2^+/P253R^* Apert syndrome mutant mice and their non-mutant littermates. Red: *Fgfr2^+/S252W^* mice; Blue: *Fgfr2^+/+^* of 252 model; Green: *Fgfr2^+/P253R^* mice; Purple: *Fgfr2^+/+^* of 253 model. Block 1: Neural crest; block 2: Mesoderm.

In contrast with the analysis considering the anatomical regionalization of the skull into facial skeleton and neurocranium that showed a simple covariation structure that could be summarized into a single PLS axis that accounted for more than 90% of the total covariation, this analysis showed that the covariation of the skull based on regions defined by neural crest and mesoderm derived structures is much more complex, and at least 4 PLS axes are needed to explain a similar amount of total covariation (PLS1: 44.9%; PLS2: 28.7%; PLS3: 13.0%; PLS4: 7.3%). The overall strength of association between the neural crest and mesoderm derived regions is significant but low (RV=0.10; p-value=0.0145), being only one third of the strength of the RV estimated between the face and the neurocranium. This indicates that these regions defined on the embryological origin of osteoprogenitor cells did not represent strongly integrated morphological units.

The PLS analyses using varying subsets of individuals including Apert syndrome mouse models and their non-mutant littermates showed that there are no substantial differences in the magnitude of association between neural crest and mesoderm derived regions when comparing the RV coefficient of both non-mutant and mutant mice, or the RV coefficient of *Fgfr2^+/S252W^*, *Fgfr2^+/P253R^* Apert syndrome mutant mice and their non-mutant littermates (Table S3).

Table S3. Results of PLS analyses before adjusting for allometry. For each grouping we provide the RV coefficient of overall integration neural crest and mesoderm derived bone and associated P-value

| **Samples used** | **RV coefficient** | **P-value** |
| --- | --- | --- |
| All groups | 0.17 | 0.0001 |
| Both NON-MUT | 0.49 | 0.032 |
| Both MUT | 0.41 | 0.024 |
| 252 NON-MUT/MUT | 0.83 | 0.001 |
| 253 NON-MUT/MUT | 0.82 | 0.001 |

Despite molecular expression analyses of the frontal and parietal bones that show significant differences in cell proliferation and osteogenic differentiation in bones derived from neural crest compared to mesoderm derived bones [3], our results suggest that complex functional/developmental constraints have a more prominent role in establishing integration patterns within the skull than the embryological origin of the cells that establish individual bones, even in the case of craniosynostosis.

**2) Endochondral/intramembranous.**

We defined two subsets of mutually exclusive landmarks based on whether the landmarks were located on bones formed by endochondral or intramembranous ossification [1,2] (Table S4).

Table S4. Landmark distribution into regions defined on the basis of the type of ossification (endochondral or intramembranous). More detailed information about the anatomical location of the landmarks can be found at <http://getahead.psu.edu/LandmarkNewVersion/P0mouseskull_updated_applet.html>

| **Code** | **Endochondral** | **Intramembranous** | **Definition** |
| --- | --- | --- | --- |
| amsph | 1 |  | Most antero-medial point on the body of the sphenoid |
| bas | 2 |  | Mid-point on the anterior margin of the foramen magnum, taken on basioccipital |
| ethma | 3 |  | Most antero-superior point of the intersection of the left and right anterior turbinates |
| locc | 4 |  | Most infero-lateral point on the squamous occipital, left |
| loci | 5 |  | Superior posterior point on the ectocranial surface of occipital lateralis on the foramen magnum, left |
| lpsh | 6 |  | Most anterior point on the anterior projection on the presphenoid, left |
| lsyn | 7 |  | Most antero-lateral point on corner of the basioccipital at the basi occipital synchondrosis, left |
| opi | 8 |  | Mid-point on the posterior margin of the foramen magnum, taken on basioccipital |
| lasph |  | 1 | Postero-medial point of the inferior portion of the alisphenoid, left |
| lflac |  | 2 | Intersection of frontal process of maxilla with frontal and lacrimal bones, left |
| lnsla |  | 3 | Most antero-medial point of the nasal bone, left |
| lpmx |  | 4 | Most infero-lateral point of the premaxillary-maxillary suture, taken on premaxilla, left |
| lpns |  | 5 | Most antero-lateral indentation at the posterior edge of the horizontal plate of the palatine bone , left |
| lpsq |  | 6 | Most posterior point on the posterior extension of the forming squamosal, left |
| lpto |  | 7 | Most postero-medial point on the parietal, left |
| lzyt |  | 8 | Intersection of zygoma with zygomatic process of temporal, taken on zygoma, left |

Using these subsets of endochondral and intramembranous-based landmarks, we performed PLS analyses pooling all mice (*Fgfr2^+/S252W^ and Fgfr2^+/P253R^* Apert syndrome mutant mice and their non-mutant littermates) before and after adjusting for allometry. The scatterplots of the PLS analyses showed that like the analysis of subsets formed on the basis of anatomical regions, the resulting MI pattern was shared between *Fgfr2^+/S252W^ and Fgfr2^+/P253R^* Apert syndrome mutant mice and between non-mutant mice (Fig. S3), with a relatively simple covariation pattern in which the first pair of PLS1 axes explained more than 90% of total covariation.

Figure S3. Scatterplot of the PLS1 scores of the regions defined by endochondral and intramembranous ossification of skull bones before (A) and after (B) removing allometry and including all *Fgfr2^+/S252W^* and *Fgfr2^+/P253R^* Apert syndrome mice and their non-mutant littermates. Red: *Fgfr2^+/S252W^* mice; Blue: *Fgfr2^+/+^* of 252 model; Green: *Fgfr2^+/P253R^* mice; Purple: *Fgfr2^+/+^* of 253 model. Block 1: Endochondral; block 2: Intramembranous.

The overall integration between these two cranial regions defined on the basis of the mode of ossification is also very high (RV=0.81), but in contrast to the results obtained for the face and the neurocranium, there are no marked differences between the magnitude of association observed in mutant and non-mutant mice (Table S5). Overall, this indicates that the endochondral and intramembraneous regions are strongly integrated but the alteration of the FGF/FGFR signaling does not alter the normal pattern of morphological integration.

Table S5. Results of PLS analyses before adjusting for allometry. For each grouping we provide the RV coefficient of overall integration between the regions based on mode of ossification and associated P-value

| **Samples used** | **RV coefficient** | **P-value** |
| --- | --- | --- |
| All groups | 0.81 | 0.0001 |
| Both NON-MUT | 0.52 | 0.016 |
| Both MUT | 0.64 | 0.001 |
| 252 NON-MUT/MUT | 0.88 | 0.001 |
| 253 NON-MUT/MUT | 0.86 | 0.001 |

**References**

1. Depew M, Tucker A, Sharpe P (2002) Craniofacial development. In: Mouse development, patterning, morphogenesis, and organogenesis. San Diego, CA: Academic Press. p. 421–498.

2. McBratney-Owen B, Iseki S, Bamforth SD, Olsen BR, Morriss-Kay GM (2008) Development and tissue origins of the mammalian cranial base. Dev Biol322: 121-132.

3. Li S, Quarto N, Longaker MT (2010) Activation of FGF signaling mediates proliferative and osteogenic differences between neural crest derived frontal and mesoderm parietal derived bone. PLoS ONE 5: e14033. doi:10.1371/journal.pone.0014033.
